# Antecedents and Moderation Effects of Maladaptive Coping Behaviors Among German University Students

**DOI:** 10.3389/fpsyg.2021.645087

**Published:** 2021-05-07

**Authors:** Lina Marie Mülder, Nicole Deci, Antonia Maria Werner, Jennifer L. Reichel, Ana Nanette Tibubos, Sebastian Heller, Markus Schäfer, Daniel Pfirrmann, Dennis Edelmann, Pavel Dietz, Manfred E. Beutel, Stephan Letzel, Thomas Rigotti

**Affiliations:** ^1^Department of Work, Organizational and Business Psychology, Institute of Psychology, Johannes Gutenberg University Mainz, Mainz, Germany; ^2^Department of Work and Organizational Psychology, Faculty of Psychology, Medical School Hamburg, Hamburg, Germany; ^3^Department of Psychosomatic Medicine and Psychotherapy, University Medical Centre, Johannes Gutenberg University Mainz, Mainz, Germany; ^4^Institute of Occupational, Social and Environmental Medicine, University Medical Centre of the University of Mainz, Mainz, Germany; ^5^Department of Communication, Johannes Gutenberg University Mainz, Mainz, Germany; ^6^Department Sport Medicine, Rehabilitation and Disease Prevention, Institute of Sport Science, Johannes Gutenberg University Mainz, Mainz, Germany; ^7^Leibniz Institute for Resilience Research, Mainz, Germany

**Keywords:** university students, stress, self-endangering behavior, quantitative demands, autonomy, presenteeism, emotion regulation, self-motivation

## Abstract

Prolonging working hours and presenteeism have been conceptualized as self-endangering coping behaviors in employees, which are related to health impairment. Drawing upon the self-regulation of behavior model, the goal achievement process, and Warr's vitamin model, we examined the antecedents and moderation effects regarding quantitative demands, autonomy, emotion regulation, and self-motivation competence of university students' self-endangering coping behaviors (showing prolonging working hours and presenteeism). Results from a cross-sectional survey of 3,546 German university students indicate that quantitative demands are positively related and autonomy has a u-shape connection with self-endangering coping. Emotion regulation was shown to be a protective factor for prolonging working hours. Moreover, self-motivation moderated the relationship between quantitative demands and prolonging of working hours, but not in the assumed direction. Self-motivation showed a systematic positive relationship with prolonging of working hours, but no relationship with presenteeism. Autonomy moderated the relationship of quantitative demands with both self-endangering behaviors. We found no moderating effects for emotion regulation of quantitative demands or autonomy and self-endangering behaviors. Besides further practical implications, the results suggest that lecturers should design their courses accordingly with less time pressure and university students should be trained in the use of autonomy.

## Introduction

With this study, we aim to study antecedents and moderators of maladaptive coping behaviors (prolonging of working hours and presenteeism) among university students. According to a recent representative study conducted by the World Health Organization in eight different countries, 31% of university students showed a mental disorder, including major depression, mania/hypomania, generalized anxiety disorder, panic disorder, alcohol use disorder, or substance use disorder (Auerbach et al., 2018). Likewise, in Germany, university students have reported high prevalence rates of depression and anxiety (Wörfel et al., 2016). Demands hindering academic achievement are important risk factors contributing to high prevalence rates of mental disorders among students (Bakker et al., 2015; Lesener et al., 2020). University students are confronted with special challenges in their university environment and due to the demands, psychological stress, and burnout, such as emotional exhaustion, are in a critical range and higher than in the general population (Stallman, 2010; Jackson et al., 2016). As we know from stress models, such as the job demands-resources model (JD-R, Demerouti et al., 2001), demands (e.g., time pressure and task complexity) can result in health impairment (Lesener et al., 2020). Clements and Kamau (2018) as well as Salmela-Aro and Upadyaya (2014), showed the health-impairment process triggered by demands also applies to demands faced by university students. Against this background, we follow Gusy et al.'s (2016) call to study how students cope with these demands and use the Study Demands-Resources (SD-R) Model as a theoretical framework (Gusy et al., 2016).

**Figure 1:**
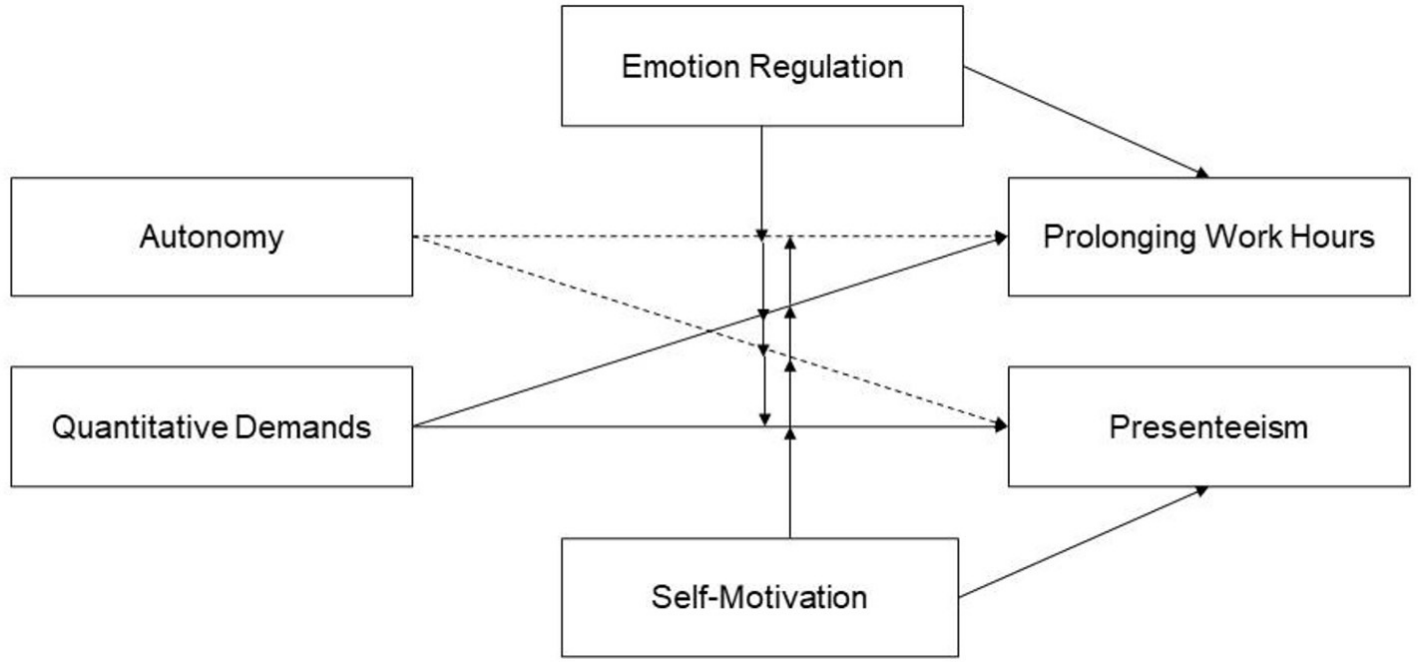
Conceptual research model.

To cope with the demands of studying and to counteract health impairments such as burnout, improving individual coping strategies and study conditions are relevant preventive approaches (Gusy et al., 2016). Coping is based on a self-regulation process and is used to deal with conditions that interfere with the goal achievement process (Carver and Scheier, 1998). There are different options to cope with stressful conditions (Dewe et al., 2010). Whereas, some of these coping strategies are functional, others could cause additional harm to an individual's health (Krause et al., 2010; Chevalier and Kaluza, 2015). Individuals differ to what extent they choose functional and dysfunctional coping strategies. Demands related to high achievement goals can promote dysfunctional coping behaviors (Baeriswyl, 2016; Baethge et al., 2019). These coping efforts can be framed as self-endangering strategies (Dettmers et al., 2016). In addition to conditional drivers, personal characteristics seem to influence the choice of coping behaviors (Carver and Connor-Smith, 2010). In this paper, we investigate how study conditions and self-regulation capacities interact in predicting the self-endangering coping behaviors of students. Furthermore, we examine the curvilinear effects of autonomy as a predictor of self-endangerment. As Warr (2017) postulated in the vitamin model, autonomy can be perceived not only as a resource but also a demand if it becomes too much and can lead to deterioration of health (Burger, 1989; Baltes et al., 2002). By investigating predictors of self-endangering coping behaviors, we add insights into the mechanism driving these effects. We focus on presenteeism and prolonging of working hours as two frequent maladaptive coping strategies and how these coping behaviors are related to students' workload and autonomy. We also integrate adaptive strategies (i.e., emotion regulation and self-motivation) as moderators in our model (see Figure 1).

Combining the SD-R model with the assumptions of the self-regulation of behavior model of Carver and Scheier (1998), our study contributes to the current coping research by (a) applying self-endangering behaviors to the student context, (b) testing curvilinear effects of autonomy on self-endangering behaviors, (c) identifying relevant conditional as well as person-related self-regulation factors that trigger self-endangering coping behaviors, and (d) by detecting such conditions and person-related self-regulation factors that could potentially buffer the use of self-endangering strategies in stressful study situations. In this study, we bring theoretical considerations developed for the employment context, as well as referring empirical evidence to a test among university students. Furthermore, a novel aspect of this study is to replace the strain outcomes as mostly used within the JD-R framework with maladaptive coping behaviors. Hence, we are suggesting coping behavior to be an important procedural aspect within the health impairment process.

We first introduce the concept of self-endangering coping behaviors and provide a rationale for the choice of our outcome variables. We will then derive hypotheses regarding the relationship of conditional aspects with self-endangering behaviors and finally introduce the moderators by referring to the self-regulation model by Carver and Scheier (1998).

### Conditional Antecedents to Self-Endangering Behavior

Self-endangering behavior is a concept developed by Peters (2011) and Krause et al. (2010). Such behaviors are defined as “actions that aim to deal with work-related demands but simultaneously increase the likelihood of health problems and impede necessary recovery from work-related stress” (Dettmers et al., 2016, p. 28). Krause et al. (2010) identified eight subscales of self-endangering behaviors: (a) extension of work time and not taking time to recover (i.e., prolonging working hours) (b) work intensification, (c) sickness presenteeism, (d) abuse of stimulants in an attempt to optimize internal states, (e) abuse of sedative substance to facilitate relaxation, (f) reducing the quality of work, (g) failure to comply with security regulations, and (h) faking. We will focus on prolonging study-related time and presenteeism because we are interested in active goal achievement behaviors that include the extension of *time* in one way or another. Prolonging working hours deals with the use of time that an individual needs for rest/recovery. Presenteeism implies an individual uses time for work rather than recuperation from illness (e.g., Krause et al., 2015).

In stressful situations, individuals can either choose an emotion-focused coping approach or a problem-focused coping approach (Lazarus and Folkman, 1984). Moreover, according to Carver and Connor-Smith (2010), coping strategies can further either be used in an active/engagement-oriented or in an avoidant/disengagement-oriented way (cf. Carver and Scheier, 1998). Referring to this coping perspective, self-endangering work behaviors may be seen as an intensified opportunity of active/engagement (problem-focused) coping when facing high demands, including the investment of internal resources (i.e., energy) to overcome obstacles (e.g., Semmer et al., 2010; Dettmers et al., 2016). These kinds of coping strategies seem to be functional at least in the short term (i.e., working longer and faster to accomplish goals), but they also drain internal resources (Dettmers et al., 2016), which might lead to a resource loss spiral with further negative effects (Hobfoll, 1989; Baethge et al., 2019).

As internal resources (i.e., energy) are restricted, coping efforts, such as self-endangering strategies, might not be beneficial in the long term (e.g., Semmer et al., 2010). Hockey (1997) pointed out that behaviors aiming at maintaining currently active goals, such as a special task or deadlines, may result in costs to emotional and physiological subsystems. He argued that “an increased commitment to task goals is assumed to imply a decrease in the relevance of other personal or biological goals, such as those concerned with leisure, rest, or well-being” (p. 78). This argument suggests that coping strategies, as prolonging working hours and sickness presenteeism, are associated with personal costs, such as health impairments (e.g., Sonnentag and Zijlstra, 2006; Semmer et al., 2010). Presenteeism can lead to lower health and the ability to work (Ferreira et al., 2019). Hansen and Andersen (2009) highlighted that presenteeism is associated with long-term sickness absence at a later date (cf. Dietz et al., 2020). Baeriswyl-Zurbriggen et al. (2014) showed that presenteeism is a maladaptive behavior, as it intensified the positive relationship between demands and burnout in a sample of teachers. A few studies have indicated that similar mechanisms can be expected for university students. Töpritz et al. (2015) reported links between university students' presenteeism behaviors and burnout, global health, and health problems. A longitudinal examination of medical students showed presenteeism leads to lower general health (Kötter et al., 2017). Céspedes et al. (2018) reported a reduction in academic performance because of presenteeism behaviors. Based on these findings, it seems particularly relevant to investigate why university students choose to use self-endangering coping behaviors, such as prolonging working hours and presenteeism. In the next section, we discuss potential antecedents to maladaptive coping behaviors.

First research findings in the work context indicate that a combination of high demands and low resources, which is viewed critically in the JD-R model, goes hand in hand with more self-endangering behavior (Miraglia and Johns, 2016; Schulthess, 2017). Employees with high quantitative demands show more self-endangering behavior (Schulthess, 2017). Schulthess (2017) also reported autonomy and goal quality to be positively related to certain aspects of self-endangering behavior. Knecht et al. (2017) showed that self-endangering work behaviors mediated the relationship between work demands and emotional exhaustion. Moreover, job demands, such as workload or physical demands, and job resources, such as autonomy, were shown to positively relate to presenteeism (Miraglia and Johns, 2016). According to Krause et al. (2015), showing self-endangering behaviors can be understood as early warning signs indicating the conditions for self-regulation are not optimal. Based on these findings, we argue, that it is important to understand conditions as antecedents of self-endangering behaviors in the context of higher education. Empirical evidence on study-related conditions as antecedents for self-endangering coping behaviors among students is scarce. Gusy et al. (2016) showed the theoretical assumptions of the JD-R model are transferable to the context of higher education. Against this background, we expect similar findings for students as in the working world. Consequently, our first hypothesis is:

*H1:* University students reporting high quantitative demands are more likely to report (a) prolonging of working hours and (b) presenteeism.

Krause et al. (2015) emphasized the role of autonomy for self-endangerment. Autonomy, which makes self-regulation possible in the first place, seems to play an ambiguous role. For a long time, autonomy was solely described as a resource triggering mainly motivational effects, which ultimately leads to higher performance and personal development (Parker et al., 2001; Bakker and Demerouti, 2017). According to Frese and Zapf (1994), autonomy can sometimes lead to unfulfillable tasks and, thus, to exhaustion. In addition to the predominantly negative connections between autonomy and burnout, the critical view of this resource is increasing (e.g., Stiglbauer and Kovacs, 2018). In line with the idea of too much of a good thing, Warr (2017) described two different types of environmental factors at work in the vitamins model. The author suggested that autonomy behaves like vitamins A and D, which are important and healthy for the body in moderate amounts but become toxic at a certain level. Several studies provide support to this claim (Burger, 1989; Baltes et al., 2002). Stiglbauer and Kovacs (2018) reported autonomy showed a u-shaped relationship to burnout, meaning burnout increased in the cases of low and high autonomy. With a medium degree of autonomy, however, less burnout occurs. Considering the curvilinear effects of autonomy on burnout, it seems plausible to also assume a curvilinear relationship with maladaptive coping behaviors. Self-endangering behaviors might be more likely to occur when there is very little or very much autonomy. For example, having little to no choice of alternatives coping strategies could lead one to go to work despite illness (presenteeism) and prolonging of working hours. Ulich and Nido (2014) showed that low autonomy is associated with higher presenteeism rates. On the other extreme, a high degree of autonomy can lead to a high degree of personal responsibility for the achievement of goals. Therefore, we expect a curvilinear relationship between autonomy and self-endangering coping behavior.

*H2:* A curvilinear relationship exists between autonomy and (a) prolonging of working hours and (b) presenteeism.

It is reasonable that, in addition to conditions, personal factors influence the self-regulatory process and should also influence the occurrence of self-endangering behaviors (Krause et al., 2015; Baeriswyl, 2016). Regarding active/engagement-focused coping, individuals need competencies to move forward in reaching goals (e.g., Carver and Connor-Smith, 2010). Thus, some personal characteristics should be more connected to the active/engagement ways or to the avoidant/disengagement ways of coping. According to Dettmers and Clauß (2018), specific self-motivation competencies are relevant for individuals who are forced to design their work on their own. A further relevant aspect is emotion regulation (Carver and Connor-Smith, 2010).

### Emotion Regulation and Self-Motivation as Antecedents to Self-Endangering Behavior

The occurrence of positive and negative feelings during the goal achievement process depends on the pace with which individuals can reduce the discrepancy between anticipation and actual goal achievement (Carver and Scheier, 1998). In Carver and Scheier's (1998) view, fast reduction of the discrepancy—a fast forward movement toward goals—should be linked to positive emotions, whereas an inadequate reduction of the discrepancy between the actual state and the target state might be connected to negative emotions. The authors also argued that negative feelings arise if the goal-directed behavior is disrupted.

Hence, having control over one's emotions (i.e., having the ability to regulate emotions during situations disrupting the goal achievement process) should be beneficial (Carver and Scheier, 1998). It can be assumed that difficulties in regulating emotions can lead to unhealthy coping strategies. Monteiro et al. (2014), for example, showed that university students with low competencies to regulate their emotions show less active/engagement problem-focused coping strategies as well as more emotion- and problem-focused disengagement strategies. Disengagement coping also includes problem avoidance strategies (Monteiro et al., 2014). Concerning the context of higher education, it can be assumed that students with low emotion regulation skills may tend to delay the fulfillment of a task. As we know from Carver et al. (Carver et al., 1989; Carver and Scheier, 1998), this could lead to even larger problems, such as additional time pressure, which could be a risk for the application of self-endangering work behaviors. Therefore, we assume the following:

*H3*: A negative relationship exists between emotion regulation and (a) prolonging of working hours and (b) presenteeism.

Another factor that may influence the active, problem-focused way of the self-regulatory process is the competence of self-motivation, which facilitates goal achievement (e.g., Dettmers and Clauß, 2018). It is reasonable that people who are more engaged and able to motivate themselves are more likely to hold on to their goals—in a reasonable time. It is to be expected, therefore, that students who increasingly demonstrate this form of self-motivation will tend to work successively to fulfill their study-related tasks. In contrast to our explanations regarding low emotion regulation skills, working successively means an individual avoids risk situations for self-endangering work behaviors, leading us to the following hypothesis:

*H4*: A negative relationship exists between self-motivation competence and (a) prolonging of working hours and (b) presenteeism.

### Interaction of Conditions and Personal Resources

So far, we have focused on conditional and personal effects on self-endangering coping behaviors in isolation. In the following, we argue that contextual and personal factors likely interact in predicting self-endangering behavior.

Individuals with low competencies to regulate negative emotions may feel obliged to extend working periods by prolonging working hours or may show presenteeism. Students facing high quantitative demands and many negative emotions will try to alleviate these emotions by choosing coping options intended to reach goals. Therefore, we predict that students will show more self-endangering behaviors if quantitative demands are high and emotion regulation is low.

*H5*: Emotion regulation moderates the positive relationship between quantitative demands and (a) prolonging of working hours and (b) presenteeism. Thus, increasing emotion regulation will result in a weaker relationship.

Concerning the interplay between autonomy and emotion regulation, we propose that high competencies to regulate emotions can help students use the wide-ranging possibilities for self-regulation that are opened up by higher degrees of autonomy. That is, individuals with high emotion regulation competencies may have an open view for expanded possible actions and be more capable of using these possible actions in stressful situations. This wider view of students with high competencies may be linked to the use of healthier strategies rather than self-endangering behaviors.

*H6*: Emotion regulation moderates the relationship between autonomy and (a) prolonging of working hours and (b) presenteeism. Thus, increasing emotion regulation will result in a stronger relationship.

To maintain task fulfillment despite disturbances (as high quantitative demands), students need high self-motivation competencies. Individuals with high self-motivation competence may have a decreased risk of finding themselves in situations in which self-endangering coping behaviors are necessary to achieve goals. Self-motivation competence should prevent students from showing self-endangering behaviors from the very beginning to maintain goals.

*H7*: Self-motivation moderates the positive relationship between quantitative demands and (a) prolonging of working hours and (b) presenteeism. Thus, increasing self-motivation results in a weaker relationship.

Further, we assume that making the best use of autonomy is dependent upon students having the competence to motivate themselves to effectively benefit from the expanded possibilities. Thus, high self-motivation competence may enable individuals to utilize these expanded available options to follow up with study-related tasks, and they will likely be less in need of any external motivational factors. This means individuals who have higher self-motivation competence may make use of their higher degrees of freedom, thereby reducing the risk of canceling recovery periods.

*H8*: Self-motivation moderates the relationship between autonomy and (a) prolonging of working hours and (b) presenteeism. Thus, increasing self-motivation results in a stronger relationship.

Based on the JD-R model (Lesener et al., 2020), and its application in the student context (SD-R), demands and resources do not just stand on their own but also interact. Resources help individuals cope with demands. Bakker et al. (2005) also found a buffering effect of autonomy on workload (quantitative demands) and emotional exhaustion. Researchers found similar boosting effects of the two factors in the outcome of work engagement (Bakker et al., 2007). This interaction effect is also reasonable for showing self-endangering behaviors, coping strategies that also result in emotional exhaustion. It remains questionable how we should apply these findings to the occurrence of maladaptive coping strategies. We argue that autonomy buffers the occurrence of self-endangerment in cases of high quantitative demands.

*H9*: Autonomy moderates the positive relationship between quantitative demands and (a) prolonging of working hours and (b) presenteeism. Thus, increasing autonomy results in a weaker relationship.

## Materials and Methods

### Data Collection and Study Design

All students of the Johannes Gutenberg-University Mainz were invited by e-mail to participate in an online questionnaire. Hence, a non-probability sampling method was employed in this study. The questionnaire was online between June and August 2019, and covered information on sociodemographic as well as study conditions, psychological resources, health-related behaviors, and health outcomes. The study protocol was approved by the ethical committee of the Medical Association of Rhineland-Palatinate (No. 2019-14336). The participants provided their informed consent to participate in this study.

### Sample

This study is based on data from the *Healthy Campus Mainz* project. We sent an invitation to an online questionnaire by e-mail to all 31,967 students of the *Johannes Gutenberg-University Mainz*. Of the university students, 4,714 completed the survey beyond the first page. After manual data cleaning according to predefined criteria, 4,351 students (13.9%) fully answered the questionnaire. In a first step, we excluded 146 Ph.D. students because they face different conditions when compared to undergraduates. Moreover, graduate students are usually employed by the university in Germany. Furthermore, and following prior studies on presenteeism, we excluded 52 participants (1.2%) who showed 50 or more days of presenteeism, as such long-term cases are likely related to chronic conditions (Gerich, 2016; Dietz et al., 2020). Missing data further reduced the sample for our analyses to 3,546 participants. The majority of the participants were female (*n* = 2,648, 71.9%) and 1,035 (28.1%) were male. Mean age was 23.6 years (*SD* = 4.2). The sample included students pursuing a bachelor's degree (*n* = 2,017, 54.3%), master's degree (*n* = 817, 22.0%), state examination (*n* = 845, 22.8%), and other special degrees (*n* = 35, 0.1%). The average time participants had already spent at the university was 6.9 semesters (*SD* = 4.5).

### Measures

*Quantitative demands* were measured with one item from the Copenhagen Psychosocial Questionnaire (COPSOQ, Nübling et al., 2006) that was adapted to the context of higher education: “Do you have enough time to complete all your study-related tasks?” Students could answer this question on a 5-point Likert scale, ranging from 1 = *never/hardly* ever to 5 = *always*. To capture quantitative demands, the item was recoded.

*Autonomy* was measured using the BARI-S questionnaire (Gusy and Lohmann, 2011). An example item is, “I can arrange my studies according to my wishes.” The scale consists of six items, which could be answered on a 6-point Likert scale ranging from 1 = *never* to 6 = *always*. Cronbach's alpha was 0.78.

*Prolonging working hours* was measured using a shortened and adapted version of the Self-Endangering Work Behavior scale (Krause et al., 2015). Our adapted scale included four items. An example item is, “I have skipped leisure time activities to work instead.” We utilized a 5-point Likert scale ranging from 1 = *rarely/never* to 5 = *very often*. The reference period was the last month, and Cronbach's alpha was 0.77.

*Presenteeism* was assessed with one item from Töpritz et al. (2015): “How many days did you work for your studies this semester (at university, at home, on an internship) even though you felt so sick and it would have been better not to do so?” Answers could be provided in an open format.

*Emotion regulation* was assessed with the cognitive reappraisal subscale of a short form of the Emotion Regulation Questionnaire (Abler and Kessler, 2009). The two items were, “If I want to feel more positive feelings (like joy or cheerfulness), I try to think about the situation differently.” and “If I want to feel less negative feelings (like sadness or anger), I try to think about the situation differently.” The questions were measured on a 7-point Likert scale ranging from 1 = *I totally disagree* to 7 = *I totally agree*. The two items correlated with *r* = 0.78 (*p* < 0.001).

*Self-motivation competence* was measured with three items developed by Dettmers and Clauß (2018). One example item is, “When you think about your studies, how well do you manage your motivation?” The items could be answered on a 5-point Likert scale ranging from 1= *not at all* to 5 = *completely*. Cronbach's alpha was 0.84.

Concerning *control variables*, previous studies show gender differences in coping behaviors (Nübling et al., 2006), prolonging working hours (Dettmers et al., 2016), and presenteeism (Miraglia and Johns, 2016; Kötter et al., 2017; Dietz et al., 2020). Moreover, subjective general health also appears to play a role in presenteeism in university students (Kötter et al., 2017). Since the individual development of students throughout the study can also affect the occurrence of self-endangering behaviors, the semester and weekly schedule were included as control variables in addition to sex and general health. General health was measured with one item developed by Nübling et al. (2006): “If you rate the best conceivable state of health with 10 and the worst conceivable with 0: How many points do you then assign for your current state of health?”

### Data Analysis

Prior to testing the hypotheses, to ensure the conceptual distinction of constructs (as shown in Figure 1), we ran a set of confirmatory factor analyses in MPlus 7.2. A six-factor model showed a better fit to the data [$\chi_{\left(138\right)}^{2}$ = 2608.23, CFI = 0.91; TLI = 0.89, RMSEA = 0.07], compared to a four-factor model, combining items of self-motivation and emotional regulation in one factor [$\chi_{\left(143\right)}^{2}$ = 4,519.88, CFI = 0.84; TLI = 0.81, RMSEA = 0.09] or a one factor model [$\chi_{\left(152\right)}^{2}$ = 17.184.78, CFI = 0.37; TLI = 0.29, RMSEA = 0.17).

Hypotheses were then tested with SPSS (Version 26) using step-wise hierarchical regression with five blocks separately for prolonging of working hours, and presenteeism as the two outcome variables. In a first step we included sex, semester, weekly schedule, and general health as control variables. In a second step we included autonomy and quantitative demands, followed by the quadratic effect of autonomy in a third step. In the fourth step emotion regulation and self-motivation (i.e., main effects of moderators) were added to the regression equation, and in the fifth, and last step all proposed interactions were added.

## Results

Descriptive statistics and bivariate correlations among the variables can be found in Table 1. In accordance with our predictions, autonomy was negatively and quantitative demands positively related to prolonging of working hours (*r* = −0.28, *p* < 0.001; *r* = 0.47, *p* < 0.001, respectively) and presenteeism (*r* = −0.12, *p* < 0.001; *r* = 0.25, *p* < 0.001, respectively). Emotion regulation and self-motivation showed significant negative but small correlations with prolonging of working hours (*r* = −0.07, *p* < 0.001; *r* = −0.04, *p* = 0.008, respectively) and presenteeism (*r* = −0.06, *p* < 0.001; *r* = −0.12, *p* < 0.001, respectively).

**Table 1:** Bivariate correlations, means, and standard deviations of study variables.

	***M***	***SD***	**1**	**2**	**3**	**4**	**5**	**6**	**7**	**8**	**9**	**10**
1. Sex^a^	–	–	–									
2. Semester	6.91	4.52	0.03	–								
3. Schedule	17.51	10.36	0.04^*^	−0.25^***^	–							
4. General health	7.48	1.70	0.01	−0.04^*^	0.06^**^	–						
5. Autonomy	2.37	0.79	0.06^**^	0.07^**^	−0.28^***^	0.03^*^	(0.78)					
6. Quantitative demands	3.45	1.18	−0.09^***^	0.03	0.19^***^	−0.16^***^	−0.30^***^	−				
7. Emotion regulation	3.86	1.62	−0.22^***^	0.02	−0.04^*^	0.13^***^	0.01	−0.02	(0.78)			
8. Self-motivation competence	3.58	0.72	−0.06^**^	0.00	0.05^**^	0.29^***^	0.10^***^	−0.24^***^	0.09^***^	(0.84)		
9. Prolonging working hours	3.20	0.88	−0.11^***^	0.03	0.22^**^	−0.15^***^	−0.28^***^	0.47^***^	−0.07^***^	−0.04^**^	(0.77)	
10. Presenteeism	4.38	6.22	−0.13^***^	0.07^***^	0.06^**^	−0.28^***^	−0.12^***^	0.25^***^	−0.06^***^	−0.12^***^	0.34^***^	–

*n = 3,606–3,714*.

a *1, male; 0, female; M, mean, SD, standard deviation; Cronbach's alphas in parantheses*.

* *p < 0.05*,

** *p < 0.01*,

*** *p < 0.001*.

Results from the regression analyses revealed that all control variables were significantly related to prolonging of work hours and presenteeism, explaining 10 and 11% of the variance, respectively. Male students reported less of both behaviors when compared to female students. The longer students spent at the university, the more likely they reported prolonging of working hours and presenteeism. Likewise, the number of weekly hours of course time was positively related to both outcomes. General health showed a negative relationship with both dependent variables (see Tables 2, 3).

**Table 2:** Stepwise regression analyses for the dependent variable prolonging working hours.

	**Prolonging of working hours**									
	**Step 1: controls**		**Step 2: conditions**		**Step 3: autonomy squared**		**Step 4: self-regulation**		**Step 5: interactions**	
	***B*(*SE*)**	**β**	***B*(*SE*)**	**β**	***B*(*SE*)**	**β**	***B*(*SE*)**	**β**	***B*(*SE*)**	**β**
Intercept	3.27(0.02)		3.24(0.02)		3.22(0.02)		3.21(0.02)		3.22(0.02)	
Sex	−0.24(0.03)	−0.12^***^	−0.14(0.03)	−0.07^***^	−0.14(0.03)	−0.07^***^	−0.13(0.03)	−0.07^***^	−0.13(0.03)	−0.07^***^
Semester	0.02(0.00)	0.09^***^	0.01(0.00)	0.06^***^	0.01(0.00)	0.06^***^	0.01(0.00)	0.05^***^	0.01(0.00)	0.05^***^
Schedule	0.02(0.00)	0.26^***^	0.01(0.00)	0.14^***^	0.01(0.00)	0.14^***^	0.01(0.00)	0.13^***^	0.01(0.00)	0.13^***^
General health	−0.08(0.01)	−0.16^***^	−0.05(0.01)	−0.09^***^	−0.05(0.01)	−0.09^***^	−0.06(0.01)	−0.11^***^	−0.05(0.01)	−0.10^***^
Autonomy (AU)			−0.14(0.02)	−0.12^***^	−0.15(0.02)	−0.13^***^	−0.15(0.02)	−0.14^***^	−0.15(0.02)	−0.14^***^
Quantitative demands (QD)			0.29(0.01)	0.39^***^	0.29(0.01)	0.38^***^	0.30(0.01)	0.40^***^	0.30(0.01)	0.40^***^
Autonomy^2^					0.04(0.02)	0.04^*^	0.04(0.02)	0.04^*^	0.02(0.02)	0.02
Emotion regulation (ER)							−0.02(0.01)	−0.03^*^	−0.02(0.01)	−0.03^*^
Self–Motivation (SM)							0.11(0.02)	0.09^***^	0.11(0.02)	0.09^***^
AUxER									0.02(0.01)	0.02
AUxSM									0.02(0.03)	0.01
QDxER									0.00(0.01)	0.01
QDxSM									0.03(0.02)	0.04^*^
QDxAU									−0.03(0.02)	−0.03^*^
Δ*R*^2^		0.098^***^		0.169^***^		0.001^*^		0.007^***^		0.003^*^

*N = 3,564*,

* *p < 0.05*,

*** *p < 0.001*.

**Table 3:** Stepwise regression analysis for the dependent variable presenteeism.

	**Presenteeism**									
	**Step 1: controls**		**Step 2: conditions**		**Step 3: autonomy squared**		**Step 4: self-regulation**		**Step 5: interactions**	
	***B*(*SE*)**	**β**	***B*(*SE*)**	**β**	***B*(*SE*)**	**β**	***B*(*SE*)**	**β**	***B*(*SE*)**	**β**
Intercept	4.85(0.11)		4.77(0.11)		4.52(0.13)		4.52(0.14)		4.55(0.14)	
Sex	−1.85(0.22)	−0.14^***^	−1.56(0.21)	−0.12^***^	−1.57(0.21)	−0.12^***^	−1.57(0.22)	−0.12^***^	−1.56(0.22)	−0.11^***^
Semester	0.12(0.02)	0.09^***^	0.10(0.02)	0.08^***^	0.10(0.02)	0.07^***^	0.10(0.02)	0.08^***^	0.11(0.02)	0.08^***^
Schedule	0.06(0.01)	0.10^***^	0.03(0.01)	0.05^**^	0.03(0.01)	0.04^*^	0.03(0.01)	0.04^*^	0.03(0.01)	0.04^*^
General health	−1.02(0.06)	−0.28^***^	−0.91(0.06)	−0.25^***^	−0.92(0.06)	−0.26^***^	−0.92(0.06)	−0.25^***^	−0.91(0.06)	−0.25^***^
Autonomy (AU)			−0.32(0.13)	−0.04^*^	−0.47(0.14)	−0.06^**^	−0.47(0.14)	−0.06^**^	−0.47(0.14)	−0.06^**^
Quantitative demands (QD)			0.86(0.09)	0.17^***^	0.85(0.09)	0.16^***^	0.84(0.09)	0.16^***^	0.82(0.09)	0.16^***^
Autonomy^2^					0.40(0.12)	0.06^**^	0.41(0.12)	0.06^**^	0.30(0.13)	0.04^*^
Emotion regulation (ER)							0.03(0.08)	0.01	0.03(0.08)	−0.01
Self–motivation (SM)							−0.08(0.15)	−0.01	−0.07(0.14)	−0.01
AUxER									−0.11(0.10)	0.01
AUxSM									0.06(0.19)	0.01
QDxER									−0.05(0.06)	−0.01
QDxSM									0.21(0.11)	0.03
QDxAU									−0.28(0.11)	−0.04^*^
Δ*R*^2^		0.109^***^		0.029^***^		0.003^**^		0.000^*^		0.002

*N = 3,564*,

* *p < 0.05*,

** *p < 0.01*,

*** *p < 0.001*.

### Direct Effects of Conditions

**Figure 2:**
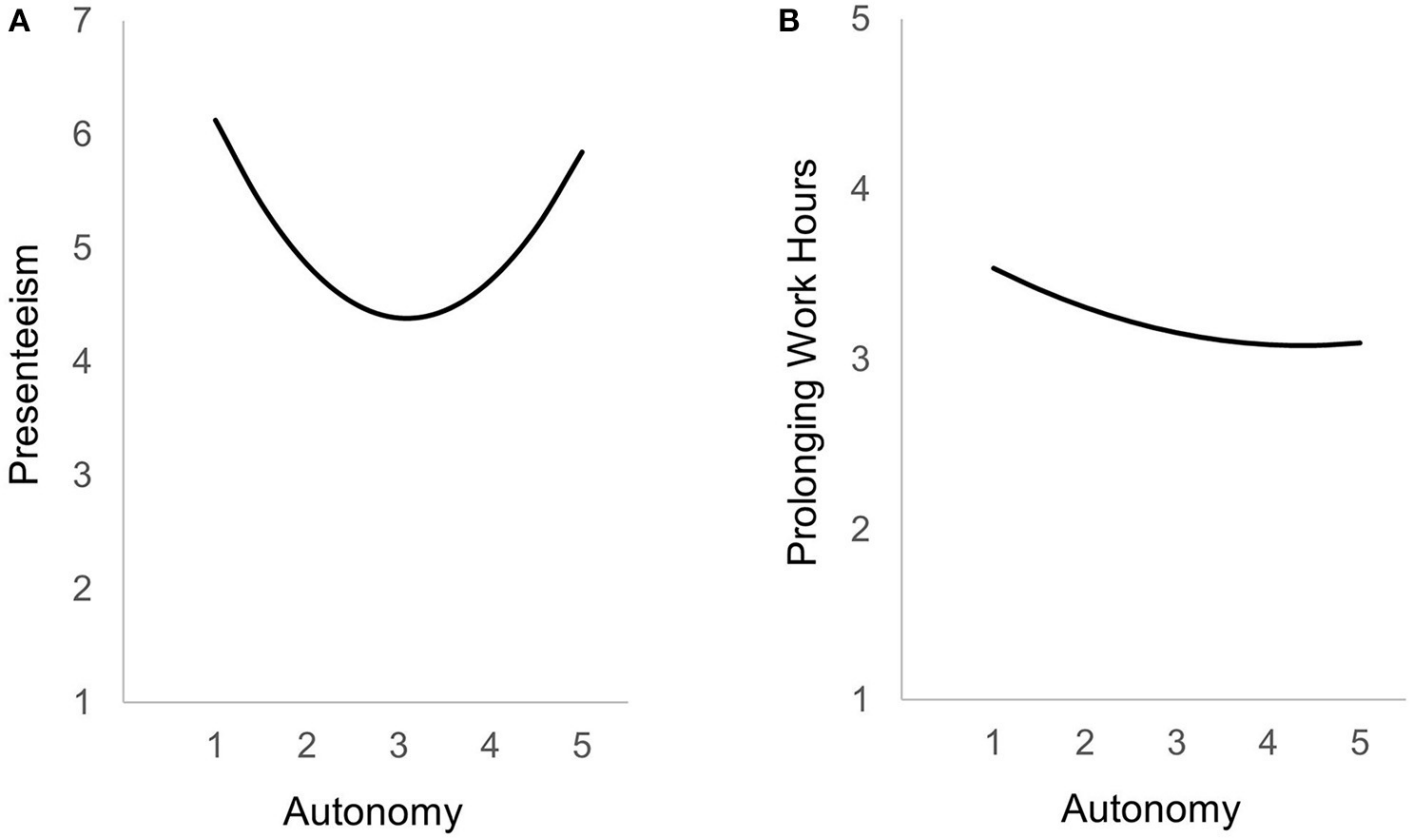
**(A)** Curvilinear effect of autonomy on presenteeism. **(B)** Curvilinear effect of autonomy on prolonging of working hours.

Quantitative demands were positively related to prolonging of working hours and presenteeism (see Tables 2, 3, Step 2), lending support to H1a and H1b. Furthermore, we predicted a curvilinear relationship of autonomy with self-endangering coping behaviors (H2a/H2b), which was supported for both dependent variables (see Tables 2, 3, Step 3). Figures 2A,B depict plots of these curvilinear relationships. There is evidence for an additional decrement effect of autonomy in predicting prolonging of working hours, even though an increase of this behavior is probably only apparent at extreme values. Concerning presenteeism the curvilinear effect as depicted in Figure 2B is u-shaped, supporting the prediction that very low, as well as very high values auf autonomy increase the likelihood to show presenteeism.

### Direct Effects of Self-Regulation

Emotion regulation showed a significant negative relationship with prolonging of working hours and presenteeism, supporting H3a and H3b. Self-motivation showed no significant direct relationship with presenteeism but, against prediction, a positive relationship with prolonging of working hours; hence, H4a and H4b must be declined.

### Interaction Effects

**Figure 3:**
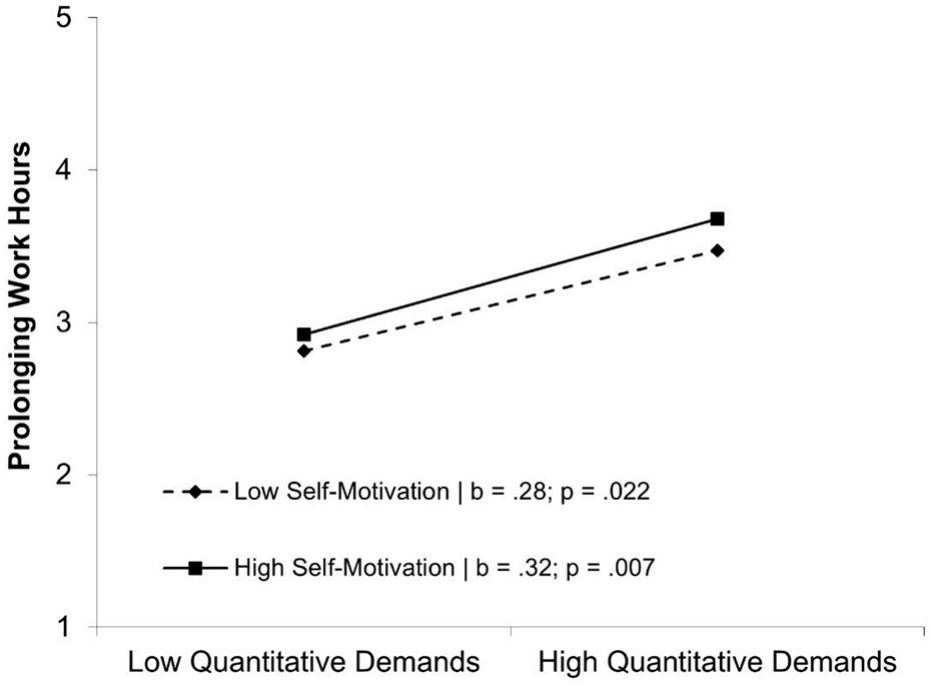
Moderating effect of self-motivation on the relationship between quantitative demands and prolonging of working hours.

**Figure 4:**
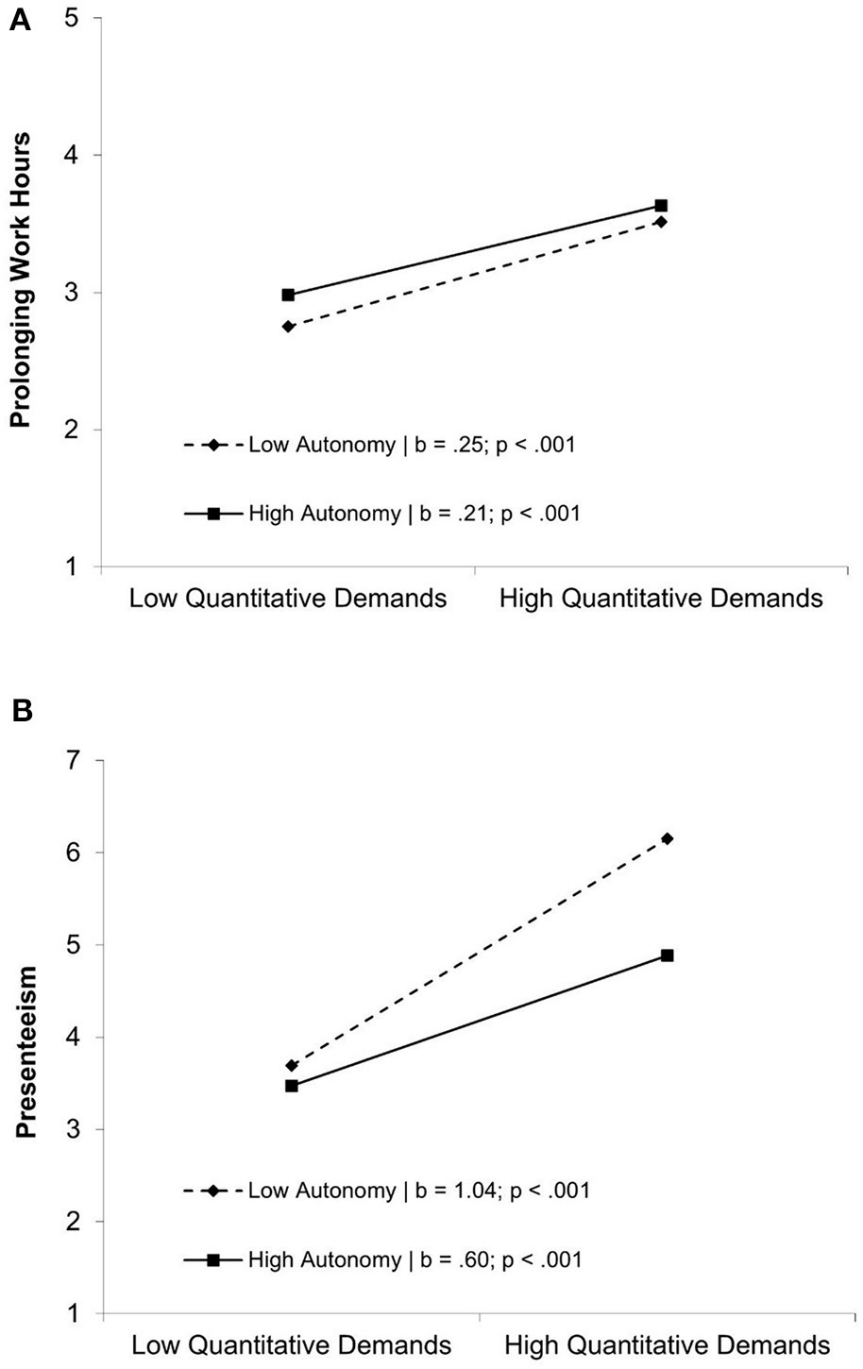
**(A)** Moderating effect of autonomy for the relationship between quantitative demands and prolonging of working hours. **(B)** Moderating effect of autonomy for the relationship between Quantitative Demands and Presenteeism.

Out of the four possible interaction effects between conditions (quantitative demands/autonomy) and self-regulation (emotion regulation/self-motivation) for each dependent variable, only one interaction was significant. Concerning the prolonging of working hours, self-motivation moderated the effect of quantitative demands (see Figure 3). A simple slope test revealed a significant positive effect for low values of self-motivation (*b* = 0.28; *p* = 0.022) as well as for high values (*b* = 0.32; *p* = 0.007). As this effect was not as predicted, we have to reject hypotheses H5–H8. We also suggested autonomy to moderate the relationship of quantitative demands with prolonging of working hours, as well as presenteeism, and we could support this assumption in our sample (see Figures 4A,B). Simple slope tests regarding prolonging working hours revealed a significant positive effect for low values of autonomy (*b* = 0.25; *p* < 0.001) as well as for high values (*b* = 0.21; *p* = < 0.001). Simple slope tests for presenteeism revealed a significant positive effect for low values of autonomy (*b* = 1.04; *p* < 0.001) as well as for high values (*b* = 0.60; *p* < 0.001). As predicted these results point toward a buffering effect of autonomy, supporting H9a, and H9b.

## Discussion

This study aimed to investigate the direct and interactional effects of conditional as well as personal predictors of self-endangering coping behaviors (i.e., prolonging of working hours and presenteeism) in a large sample of university students. Our results support the assumptions that quantitative demands are positively and autonomy is negatively related to self-endangering behaviors. Additionally, and in line with other studies, we found support for a curvilinear effect of autonomy. In accordance with Warr's (2017) vitamin model, autonomy can be seen as an additional decrement resource. For prolonging of working hours the curvilinear effects supports a decelerating effect. For presenteeism we found clearer evidence of undesired effects for high values of autonomy. Emotion regulation proved to be a valid protection factor only for prolonging of working hours. Against predictions, self-motivation in isolation showed a systematic relationship to prolonging of working hours but no relationship with presenteeism. The two self-regulation strategies neither buffered nor boosted the relationships of autonomy with self-endangering behaviors. In contrast to our prediction, self-motivation boosted the effect of quantitative demands on prolonging working hours.

Our results supporting H1a and H1b, assuming quantitative demands are associated with prolonging working hours and presenteeism, are in line with the scientific evidence gathered in the working context (Bakker et al., 2015; Baeriswyl, 2016). Research in the employment context showed, that presenteeism relates positively with quantitative demands (Baeriswyl, 2016). In other words, when there are high demands in their studies due to many tasks, time pressure, and deadlines, university students tend to give up free time for their study-related tasks and they tend to study or attend lectures or seminars despite being ill. As suggested in H2a and H2b the curvilinear effect of autonomy, as previously reported for samples of employees (Burger, 1989; Baltes et al., 2002) could be replicated for the study context and even showed to apply to self-endangering behaviors. These findings indicate that a medium level of autonomy for students seems to be health-promoting for self-care, but that very low and very high levels of autonomy promote maladaptive coping behaviors of prolonging working hours, and especially for presenteeism.

Regarding H3a and H3b, only a negative link of emotion regulation with prolonging working hours was found, which means that students with high emotion regulation tend to show less prolonging working hours but not more or less presenteeism. This is partly in line with the propositions of Carver and Scheier (1998) and other studies in this area (Gross and Feldman Barrett, 2011; Monteiro et al., 2014).

Our predictions that lower self-motivation competence is positively associated with prolonging working hours and presenteeism (H4a/H4b) was not supported. Surprisingly, the effect showed to be in the opposite direction: High self-motivation was positively associated with prolonging working hours. These findings are in contrast with past work; up to now, experts assumed self-motivation related negatively to psychological strain (Dettmers and Clauß, 2018). This finding suggests that behind the positive motivational and health-promoting effect, too much self-motivation may cause students to overextend themselves. This aspect of expanding for goals could, for example, be a precursor or behavior before the appearance of burnout symptoms, since the constant extension of working hours as well as working even though one is sick could lead to such a disease in the long run. The link of self-motivation with maladaptive coping strategies and associated health impairments should be considered in more detail in the future.

Contrary to our expectations, we could not confirm emotion regulation as a moderator between study condition and self-endangering behaviors, as suggested in H5a, H5b, H6a, and H6b. These findings contradict previous studies of the roles of emotion regulation (Gross and Feldman Barrett, 2011; Monteiro et al., 2014). Yet other studies have not addressed the specific context of university students, and thus emotion regulation might be less relevant as a buffering aspect among students, as among employees. Regarding H7a, we found a boosting effect of self-motivation competence on the relationship between quantitative demands and prolonging working hours, which was contrary to our expectations. In other words, when university students rate their quantitative demands as high, a high self-motivation competence is associated with even more prolonging of working hours. Showing this harmful behavior in connection with previous positively associated competencies (Bredehöft et al., 2015; Dettmers and Clauß, 2018) is a new insight that deserves further attention in the future. Up to now, self-motivation had been viewed as a positive aspect, which should be reinforced. One possible explanation for the positive relationship of self-motivation and self-endangering behaviors is that with a high degree of self-motivation competence students tend to be overcommitted to their studies (Siegrist, 2002). Students could show an excessive willingness to expend energy and, therefore, show more prolonging of working hours. Previous studies on presenteeism also show that employees with high levels of presenteeism tend to be overcommitted (Hansen and Andersen, 2008; Cicei et al., 2013), but no connection was made with high self-motivation competence or prolonging of working hours. Thus, if students are good at motivating themselves and then face high quantitative demands, they will engage in more self-endangering behaviors than usual. These findings warrant reevaluation of the recommendation to increase self-motivation competencies. If these findings can be replicated in future research, it would suggest that self-motivation competence in performance situations should be viewed with caution and not fostered to their limits. As soon as people show more prolonging of working hours, this poses a risk for their health and well-being (Hansen and Andersen, 2009; Baeriswyl, 2016). Habits that build up during one's university education are likely to spill over to coping behaviors applied later in the working context; thus, preventing maladaptive coping among students is an important prevention strategy. If such behavior occurs more frequently in the professional world and no attempt is made to prevent it, mental health is at risk. Concerning the small effect sizes, we must be cautious in deferring practical implications, especially because no significant moderation effect of self-motivation competence on the relationship of quantitative demands on presenteeism could be observed.

The present study found no evidence that self-motivation competence moderates the relationship between autonomy and self-endangering behaviors (H8a/H8b). The theoretical reasoning that self-motivation helps in making the best use of autonomy needs to be refined for student populations. Apart from our predominant focus on the role of self-regulation, our study presents a perspective on the moderating role of autonomy on the relationship between quantitative demands and prolonging working hours (H9a) and presenteeism (H9b). As presumed, our results show a buffering effect of autonomy. Hence, we were able to transfer previous evidence in the employment context (Monteiro et al., 2014; Jackson et al., 2016; Dettmers and Clauß, 2018) to a sample of university students.

### Theoretical Implications

We showed that students, like employees, engage in both self-endangering behaviors prolonging working hours and presenteeism and that this behavior are related to demands and resources. To preventively address the negative health effects, we examined the predictors of self-endangering behaviors and included conditions, as well as personal characteristics of self-regulation. Our study extended the understanding of the SD-R model regarding the aspect of coping (Baeriswyl, 2016; Bakker and Demerouti, 2017). We further combined the SD-R model with the self-regulation concepts of Carver and Scheier (1998) to understand the role of competencies in the goal-achieving context. We identified relevant conditional as well as person-related self-regulation factors that trigger self-endangering coping behaviors. We showed that quantitative demands are related to more self-endangering behaviors and autonomy is related to less self-endangering behaviors.

The results support Bakker and Demerouti (2017) proposal to extend the JD-R theory. Emotion regulation was found to be a protective factor, supporting the linkage of JD-R theory or SD-R theory with self-regulation theory to self-endangering behavior. Thus, the ability to regulate emotions seems to prevent the use of maladaptive coping strategies. A preventive effect of emotion regulation (reappraisal) with burnout has already been reported in the work context (Zhao et al., 2019; Chang, 2020). Our study suggests that coping processes and personal resources should be considered together with demands and resources to understand processes that are harmful to health (cf. Baeriswyl, 2016). Consequently, special attention should be paid to the function and effect of self-motivation competence in the performance context, as there appear to be potential negative side effects. The understanding of the effect of self-motivation competencies was sharpened to a certain extent since these competencies can act as a booster for showing self-endangering coping behaviors. Of particular importance is also the interaction effect, which indicates that quantitative demands lead to a renunciation of leisure time activities in favor of studying if students can motivate themselves very well. This combination of characteristics should be taken up in the future, both in the student and in the work context, to strengthen health-promoting behaviors and not to force students to act in such a way. Finally, we highlighted that autonomy does not show a linear function in its relationship to self-endangering coping behaviors. As expected, the considerations to extend Warr's (2017) vitamin model could be confirmed. These significant interaction effects support the assumption that only a certain level of autonomy for students should be considered beneficial to health. The associated theoretical considerations for the occurrence of the maladaptive coping styles prolonging working hours and presenteeism would still need to be confirmed in further studies, but the design of study conditions should also take on greater importance apart from work design. This is important because if students are overburdened with too much autonomy early on in their learning environment, these maladaptive coping strategies could become entrenched and occur in their working lives as well. A health-oriented behavior of our future leaders of society should be supported. Additionally, besides the consideration of study conditions, the provision of alternative coping strategies of, for example, quantitative demands would be appropriate.

### Future Research

Our results hint that the interplay of conditional and personal factors might be more complex. Emotion regulation and self-motivation competence can lead to more motivation and better job performance. However, as a health-damaging behavior, emotion regulation may also have an impact on job strain. These results deserve further investigation and explanatory approaches. An examination of self-motivation competence would also be relevant, as it may be associated with maladaptive perfectionism when considering self-endangerment behaviors. The interplay of conditional and personal factors should be further explored in the future. Other (mal)adaptive coping strategies beyond self-endangering behaviors can be considered. Competencies, such as planning (Dettmers and Clauß, 2018), could be negative predictors of self-endangerment. The understanding of autonomy and self-endangering behaviors could be expanded in future studies by exploring different facets of autonomy as well as other aspects of coping behaviors depending on the demands.

### Limitations

The following limitations should be considered when interpreting the results. Common method bias (Podsakoff, 2003) may have influenced the relationship between demands and self-endangering behaviors. Since the selection of participants was based on non-probability sampling, we cannot claim to have a representative sample. In future studies, an additional external measurement of demands could help to avoid this problem. Moreover, the data were only collected in a cross-sectional study, which does not allow to draw causal conclusions. We focused only on a few facets of self-endangering behaviors, so these results only apply to the facets of prolonging working hours and presenteeism. Our focus was on the antecedents since the health consequences of self-endangering behaviors in the work context have been sufficiently investigated. As we designed the questionnaire to provide data on many health-related aspects, we opted for short measures to sustain the compliance of our respondents.

### Practical Implications

Our investigation suggests that quantitative demands show a linear and autonomy a curvilinear relationship to self-endangering behaviors. Therefore, we recommend that student schedules and the organization of seminars and lectures be organized in a way that reduces quantitative demands and provides an optimum level of autonomy. The quantitative demands should be at a feasible level and autonomy should be in the middle range. Lecturers should rethink their courses in this regard and be trained in how to tailor requirements correctly and support the health-promoting use of resources (Bredehöft et al., 2015; Dettmers and Clauß, 2018). To avoid the appearance of presenteeism among university students in the future, it is, according to our knowledge, advisable to increase autonomy in the case of high quantitative demands. Students could be provided with examples of how to use their autonomy in a health-promoting way. Planning competence could also play a role in this respect (Dettmers and Clauß, 2018). Further, evaluated interventions on coping skills (Yusufov et al., 2019) should be offered more widely at universities.

Particular attention should be paid to the fact that, in performance situations, emotion regulation and self-motivation competence should be discouraged. This applies not only to the university context but also to the world of work and schools. Students should be made aware of the benefits of taking good care of themselves and being able to have good recovery from work (Sonnentag and Fritz, 2015). In contrast, it is important students know that prolonging working hours and presenteeism may inhibit recovery processes (cf. Krause et al., 2015).

### Conclusion

Our study contributes to the current coping research by investigating conditional and personal characteristics in relation to self-endangering behaviors. We offer further evidence for the idea that the JD-R model (or SD-R in the student context) should be extended to include the aspect of coping (cf. Baeriswyl, 2016). Our findings suggest that quantitative demands and autonomy are important factors in predicting self-endangering coping behaviors. Moreover, self-motivation can boost the effect of quantitative demands on prolonging working hours. We encourage future research to investigate further antecedents that lead to self-endangering coping behaviors to prevent health impairments of students and employees.

## Data Availability Statement

The raw data supporting the conclusions of this article will be made available by the authors, without undue reservation.

## Ethics Statement

The studies involving human participants were reviewed and approved by Ethical committees of the Medical Association of Rhineland-Palatinate (No. 2019-14336). The patients/participants provided their written informed consent to participate in this study.

## Author Contributions

All authors listed have made a substantial, direct and intellectual contribution to the work and approved it for publication.

## Conflict of Interest

The authors declare that the research was conducted in the absence of any commercial or financial relationships that could be construed as a potential conflict of interest.
